# A grey literature review of special events for promoting cancer screenings

**DOI:** 10.1186/1471-2407-14-454

**Published:** 2014-06-19

**Authors:** Cam Escoffery, Kirsten C Rodgers, Michelle C Kegler, Mary Ayala, Erika Pinsker, Regine Haardörfer

**Affiliations:** 1 Department of Behavioral Sciences and Health Education, Rollins School of Public Health, 1518 Clifton Road, 5th Floor, Atlanta, GA 30322, USA; 2 University of Minnesota, Minneapolis, MN, USA

**Keywords:** Cancer screening, Community awareness, Cancer education, Breast cancer, Colorectal cancer, Cervical cancer

## Abstract

**Background:**

Cancer remains the second leading cause of mortality in the United States. Special events such as health fairs, screening days or cultural festivals are employed often for community education about cancer screening. A previous systematic review of the published literature was conducted in 2012-2013. The purpose of this study was to conduct a grey literature component of special events that promote breast, cervical and colorectal cancer screening in the U.S.

**Methods:**

We conducted a grey literature search of dissertations/theses and conference abstracts. The theses/dissertations were restricted to those: 1) written in English, 2) published from January 1990 to December 2011, 3) examined at least one of the predefined categories of special events, 4) involved cancer screening for breast, cervical, and/or colorectal cancer, 5) included outcome data, and 6) conducted in the United States. A review of U.S. public health and cancer conference abstracts, that were readily available and had focused on at least of 3 cancer types and included outcome data, was conducted. Data were abstracted on the purpose, location, primary audience(s), activities conducted, screening provided onsite or referrals, and evaluation results.

**Results:**

The grey literature review found 6 special events. The types of events found added to the numbers found in the systematic review, especially receptions or parties and cultural festivals/events. All focused on increasing breast and cervical cancer screening except one that focused on breast cancer only. The reach of these events was targeted at mostly minorities or underserved communities. Common evidence-based strategies were group education, small media, and reducing structural barriers. Group education involved presentations from physicians, lay-health advisors, or cancer survivors, while reducing structural barriers included activities such as providing screening appointment sign-ups at the event or providing transportation for event participants. Mammogram screening rates ranged from 6.8% to 60% and Pap tests from 52% to 70%.

**Conclusions:**

Further evaluation of special events to promote cancer screening will prove their effectiveness. A grey literature review can augment a systematic review of published literature. Additional data about these events through the grey literature offered additional insights into the goals, intervention components and outcomes of interventions.

## Background

Cancer remains the second leading cause of mortality in the United States. The estimated lifetime risk of developing cancer is 45% among men and 38% among women [1]. Breast cancer accounts for nearly 30% of all new cancers diagnosed in females with 234,850 new cases expected in 2013. Colorectal cancer is the third most common cancer in both females and males and 142,820 new cases are expected to be diagnosed in 2013 [1]. Although cervical cancer leads to far fewer deaths, 12,340 women are expected to be diagnosed with it in 2013. Combined, these cancers are expected to result in 94,890 deaths, the majority of which can be prevented through regular screening tests that allow for the detection and removal of precancerous growths [1]. Cancer screening is essential to finding cancer before symptoms appear and helps with treatment or curing it when found early. According to the 2010 NHIS, 72.4% of women have been screened for breast cancer and 83.0% for cervical cancer [2]. For colorectal cancer, only 58.6% of adults ages 50 to 75 are up to date with screening [2]. These rates are below the Health People 2020 targets for screening.

The practice of cancer screening has increased due to the accessibility and affordability of the screenings [1-4]. However, there is still a disproportionate number of uninsured or underinsured individuals that do not have access to regular cancer screenings and suffer higher rates of mortality as a result [4]. Additional barriers to cancer screening include lack of knowledge, lack of a doctor’s visit in the past year or regular provider, lack of physician recommendation, no family history, or having no symptoms [5-7].

Intervention strategies such as educational workshops, mass marketing, small media, and more recently social media can increase the number of people who regularly receive cancer screenings [8]. The U.S. Preventive Task Force’s Community Guide for Preventive Services recommends intervention strategies to increase screening for breast, cervical and colorectal cancer. The Community Guide recommends provider assessment and feedback; provider and client reminders; small media (print materials) and one-on-one education to increase the uptake of breast, cervical, and colorectal cancer (FOBT) screening [8,9]. Sufficient evidence suggests that the uptake of screening for colorectal (FOBT) and breast cancer is also increased by reducing structural barriers to screening; however, these interventions need to be further examined for cervical cancer screening.

Special events such as cultural events, charity walks/runs, receptions/parties, and health fairs are routinely conducted by state health departments and community-based organizations to disseminate health promotion activities directly to the community. In a recent systematic review, ten studies that evaluated special events aimed to increase breast, cervical, and colorectal cancer screenings were described [10]. The review of 10 published events found five common types of special events: health fairs, parties, cultural events, special days, and plays. The most frequent activities mapped onto Community Guide strategies were reducing structural barriers to screening, one-on-one or group education, and provision of cancer educational materials. Screening rates as a result of the special events varied by type of screening: 1.7% to 88% of participants for mammograms, 3.9% to 10.6% for pap testing, 29.4% to 76% for FOBT and 1% to 100% for sigmoidoscopy. The special events that offered onsite screenings reported higher screening outcomes [10].

Due to the limited peer-reviewed literature on special events that aim to increase cancer screenings, a review of the grey literature was conducted. Grey literature is defined as a range of published and unpublished materials, which are not normally identifiable through conventional methods of bibliographic control [11]. It can include book chapters, books, conference abstracts, reports, unpublished data, dissertations, policy documents and personal correspondence [12]. A comprehensive review of both the peer-reviewed and non-peer-reviewed published and unpublished knowledge-base is essential in making decisions about intervention practice and effectiveness [13]. The Cochrane Handbook for Systematic Reviews suggests that 30% of the evidence-base is derived from unpublished or non-peer reviewed literature [13]. Fewer than half of trials presented at conferences continue to full publication and there are systematic differences between the trials that are published and those that are not [14-16].

Due to the labor-intensive process of searching grey literature, it is often not performed. In many cases, results found in the grey literature are not followed by a formal publication and are not disseminated at all to the public health audience [17]. In addition, grey literature has not gone through the level of rigor that peer-reviewed, published materials have; therefore, debate does exist on how much value this evidence adds to the field [12]. However, it can identify emerging evidence before a formal publication is produced and can provide fuller range of outcomes given that null results are less likely to appear in the peer-review literature.

The purpose of this research was to conduct a review of grey literature in order to identify special events for increasing breast, cervical, and colorectal cancer screening among conference proceedings and dissertations/theses. These findings will augment a systematic review of similar special events found in the published literature by examining special event interventions that were performed but have not yet been published in the peer-reviewed literature.

## Methods

### General framework for the grey literature review

The grey literature search focused on two main areas: dissertations/theses and scientific conferences. It was guided by our earlier developed conceptual model that hypothesizes relationships between special events, determinants of cancer screening behavior, and actual screening behavior [10]. Hence, the search aimed to identify literature that addressed at least one of two primary outcomes of special events including: screening determinants (e.g., increased awareness, knowledge, intentions to get screened, and referrals for screening) and completed cancer screenings.

An advisory committee that consisted of cancer researchers, funders, and practitioners from the Centers for Disease Control and Prevention (CDC), Comprehensive Cancer, Colorectal Cancer Control Program and Division of Cancer Prevention and Control, and the Cancer Prevention and Control Research Network was formed to guide the review process. The committee provided scientific input and advice on the grey literature review methods, the conceptual framework, and the inclusion criteria for non-peer reviewed/unpublished literature. The committee also played an integral role in identifying national, regional, and local cancer prevention conferences and providing conference agendas and materials when needed. The grey literature review was designated as exempt from IRB review from Emory University since it did not involve human subjects.

### Dissertation/thesis literature search

#### Types of dissertation/thesis studies

The dissertations or theses were restricted to those: 1) written in English, 2) published from January 1990 to December 2011, 3) examined at least one of the predefined categories of special events, 4) involved cancer screening for breast, cervical, and/or colorectal cancer, 5) included outcome data, and 6) conducted in the United States. Theses or dissertations were excluded if they did not report on screening determinants (e.g., intentions to get screened, appointments) or health outcomes; if the type of cancer or outcomes were not related to breast, cervical or colorectal cancer screening or if this was not apparent through reviewing the full thesis or dissertation; or if the dissertation was unable to be located through ProQuest.

#### Search methods for dissertation/thesis studies

In August 2011, the study team searched the ProQuest Database. ProQuest is the online database that houses searchable bodies of work that have been submitted by masters and doctoral students for fulfillment of a terminal degree. Keywords for the search included: *health fair, cultural event/festival, charity walk/run/walkathon, reception/dinner/gala, play, contest, and art/photo exhibit.* These terms were combined with *cancer prevention and control; breast, cervical, and colorectal cancer screening; evaluation; and cancer screening.* The search terms were developed based on PubMed MeSH headings and through consultation with a certified health sciences librarian. The resulting composite library was then exported into an Excel file for documentation of the abstract review process.

#### Identifying dissertation/thesis studies

**Figure 1:**
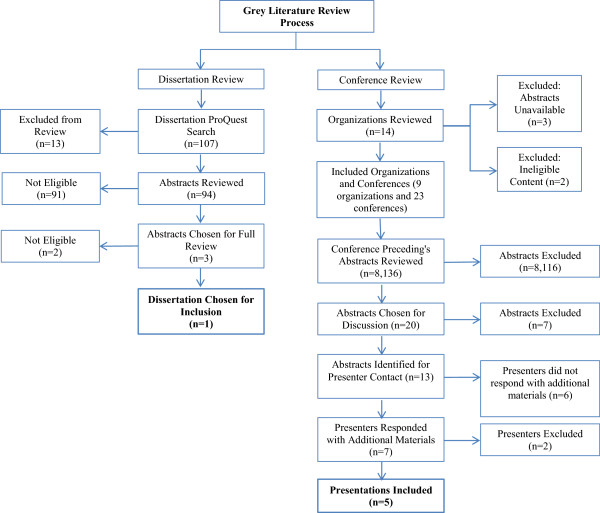
Flow diagram of study selection flow chart.

The study selection process is illustrated in Figure 1. Two reviewers independently screened the title and abstracts of all citations using an Eligibility Assessment Checklist. Abstracts were classified as relevant, potentially relevant, or not relevant. Relevant abstracts were selected for a full review of the thesis or dissertation. Abstracts of potentially relevant work were further examined independently by the two reviewers. Abstracts that did not provide enough information on outcomes to determine eligibility were included for further review. Full texts were obtained of the abstracts that met the eligibility criteria, these were read and reviewed in-full and re-examined for the eligibility criteria. Data extraction was performed independently by two reviewers. Collected data include purpose of the event, host and location of the event, primary audience, partners involved, activities conducted, screening provided onsite or referrals, and evaluation results.

#### Dissertations and theses search results

The ProQuest™ dissertations and theses search identified 107 abstracts. Of these, the study team screened 94 abstracts for eligibility since 13 had been removed as duplicates. There were 91 abstracts that did not meet eligibility criteria and were excluded from further review; common reasons were that they did not fit study definitions of special events, did not focus on breast, cervical, or colorectal cancer screening, or they did not evaluate the event. Three abstracts were selected for full-text review, and of those, 1 dissertation met the eligibility criteria outlined for the study.

## Results

### Dissertations and Theses

#### Description of dissertation study

A summary of the dissertation is provided in Table 1. The dissertation study examined an evaluation of a play that was conducted at four preschools in San Francisco, California [17]. The innovative approach of using theatre to increase knowledge and attitudes about breast cancer screening was assessed through a pre-, post-test onsite survey of the immigrant, Chinese, women-only audience. The 167 Chinese women were predominantly mothers, grandmothers, and aunts of the preschoolers performing, ranged from ages 25-77 (Mean = 40), and varied in the number of years that they had lived in the United States. Women reported emigrating from China (53.8%) or Hong Kong, Macua, and Taiwan (17.9%).

**Table 1:** Special events grey literature summary table

**Author source**	**Presentation title & focus**	**Data collection**	**Event participants**	**Special event description**	**Results**
**Conferences – Single Component (Special event only)**					
K. White [19]	Does conducting routine community health outreach events reach new women? An evaluation of a breast & cervical cancer screening program for Latina immigrants	Pre-event and Post-event	Target audience: Latina immigrants	Event Type: Educational Luncheon	Baseline Screening:
Conference:	Cancer focus: Breast and Cervical		Sample: N = 932 Latina immigrants; age: 70.7% 18-39, 19.4% 40-49, and 9.9% 50-88; 6.9% Insured	Description: Educational luncheons focused on breast and cervical cancer, were hosted at local churches on Saturday mornings to increase screening among target population.	No data available
APHA Conference, 2010				Event Components:	Post Event Screening:
				• Educational presentations from Spanish-speaking physicians and/or breast cancer survivors	Pap Tests: 412 (52%)
				• Referrals and ability to schedule pap tests and mammograms	Out of n = 792, women who completed information records
				• Magnets were provided with appointment and provider information for the low-cost pap and free mammogram screenings	Mammogram: 141 (60.8%)
				Community Guide Strategies: GE, CRR, SM, ROPC, RSB	Out of n = 232, women who completed information records and were over 40 years of age
					Other Outcomes: No data available
					Cost: No data available
C. Rice [20]	Implementation of an Evidence-based Cancer Prevention and Control Program through Texas AgriLife Extension: Friend to Friend	Pre-event and Post-event	Target audience: Uninsured and underserved women in rural Texas	Event Type: Health Party	Baseline Screening:
Conference:	Cancer focus: Breast and Cervical		Sample: N = 1,095 participants; 41% under age 50, 32% 50-64, and 27% 65 and older; 93% white, 7% African American	Description: 45 health parties were planned throughout 40 counties. The parties were designed for adult women to hear a health professional speak and to identify clinics that provide uninsured women screening.	Mammogram Status:
CPRIT, 2011				Event Components:	Within last 12 months: 49.4%
				• Group educational sessions were provided by health care providers or cancer survivors; followed by small group discussions	Within last 24 months: 10.1%
				• Educational materials were provided in English and Spanish	Within the last 3-years: 5.4%
				• Incentives were provided	Longer than 3 years: 10.0%
				• One party provided mammogram screening on-site	Never had a mammogram: 25.2%
				• Providers came to the events to sign participants up for screening referrals and scheduling of appointments	Pap Test Status:
				• Community Guide Strategies: GE, SM, RSB, ROPC	Within last 12 months: 54.3%
					Within last 24 months: 15.6%
					Within last 3 years: 5.1%
					Longer than 3-years: 20.9%
					Never had a Pap: 4%
					Post Event Screening: No data available
					Other outcomes: Knowledge
					Cost: No data available
D. Dahlke [22]	Increasing Screening Rates for Latinas Using Health Fiestas and Promotoras	Pre-event and Post-event	Target audience: Underserved Latinas	Event Type: Cultural Event/Health Fiesta	Baseline Screening:
Conference:	Cancer focus: Breast and Cervical		Sample: N = 8,026 (for the Georgia Events); 86% uninsured	Description: The health fiestas were designed to reduce health disparities within the Latino Community through the events and a patient navigation program which were designed to provide access to early detection and screening opportunities.	Mammogram status:
CPRIT, 2011				Event Components:	In the last 12 months: 4,816 (60%)
				• Educational materials were provided in Spanish	Pap Test status:
				• Food, music, dancing, and fun activities were provided	Last 12 month: 6,423 (80%)
				• Local health care providers offered free or low-cost screenings	Post Event Screening:
				• Community Guide Strategies: 1 on 1, GE, SM, RSB, ROPC	Mammogram: 218 (6.8%) of screening eligible
					Clinical Breast Exams: 3158 (39.3%)
					Other outcomes: Barriers to care
					Cost: No data available
**Dissertation – Single Component**					
A. Sun [17]	Promoting Breast Cancer Screening Among Chinese American Women Through Young Children’s Theatrical Performance, 2009	Pre-event and Post-event	Target audience: Chinese American females, able to read either Chinese or English, 18 or older, and audience members	Event Type: Play	Baseline Screening:
*Dissertation Source:* Proquest	Cancer focus: Breast		Sample: 173 participants; Average age: 40.1; 100% Asian and female; 85% covered by insurance	Description: a theatrical pre-school performance in educating Chinese American women about breast cancer detection.	Had a mammogram in past year (women > 40 years old):
				Event Components:	34 (55.7%)
				• Pre-school theatrical performances were used as an screening educational tool	Change in Knowledge Score Pretest to Posttest:
				• Foam boards in the play displayed Susan G. Komen guidelines	10.2% decreased
				Community Guide Strategies: SM, GE	41.8% had no change
					48.0% increased
					Post Event Screening:
					No data available
					Other outcomes: Attentiveness, Acculturation
					Cost: No data available
**Conferences – Multiple Components (Special event combined with other community events)**					
L. Vera-Cala [21]	Effectiveness of Cuidandome (Taking Care of Me)	Pre-event and Post-event	Target audience: Latina-immigrant women in Dane County, WI	Event Type: Health Parties	Baseline Screening:
Conference:	Cancer focus: Breast and Cervical		Sample: N = 1,381; Spanish speaking women	Description: 167 home health parties led by lay health advisors were performed to increase breast and cervical cancer screening among Latino-immigrant women.	Mammogram (n = 222):
APHA Conference, 2011				Event Components:	73 (33%)
				• One hour educational session on breast and cervical cancer	Pap Test (n = 222):
				• Same language lay health providers used to promote cervical and breast cancer screening	135 (61%)
				• Provided resources to party participants on how to apply for free or reduced cost screening	Post Event Screening:
				Community Guide Strategies: GE, SM, MM, RSB, ROPC	Mammogram:
					1-month post: 91 (41%)
					3-months post: 127 (57%)
					15-months post: 118 (53%)
					Pap Test:
					1-month post: 151 (68%)
					3-months post: 155 (70%)
					15-months post: 140 (63%)
					Other outcomes: Intention, Knowledge
					Cost: Approximately $130.00 per event
B. Hunt [18]	Reaching at-Risk Women to Promote Breast Cancer Screening on the Westside of Chicago	Pre-event and Post-event	Target audience: Women over 40, African American and Hispanic, uninsured or under insured women	Event Type: Forums and Health Fairs	Baseline Screening:
Conference:	Cancer focus: Breast and Cervical		Sample: N = 880; 87% over 40; 58% African American; 36% Mexican or Puerto Rican; 40% no insurance	Description: The forums and health fairs were designed to reduce disparities and improve overall breast health outcomes by educating, engaging, and empowering women to increase routine mammogram utilization and to share their knowledge.	Mammography (women > 40):
APHA Conference, 2011				Event Components:	Within 2 years: 443 (61%)
				• Community health workers provided 45-minute educational presentations and helped participants set-up appointments	2 or more years ago: 187 (26%)
				• Client reminders are sent to participants when due for their next screening	Never: 95 (13%)
				• Gifts, raffles, and specialized cookies were provided as incentives	Post Event Screening:
				• Transportation was provided	Mammography: n = 357 (40.1%)
				• Women requesting screening appointments are assigned patient navigator.	Cost: $3,000-$4,000 per forum
				Community Guide Strategies: GE, CRR, SM, RSB	

Note. Community Guide recommended strategies abbreviations: *CRR* Client Reminders and Recalls, *CI* Client Incentives, *ROPC* Reducing Out-of-Pocket Costs, *GE* Group Education, *1 on 1* = One-on-One Education, *MM* Mass Media, *SM* Small Media, *RSB* Reducing Structural Barriers.

The twenty minute play used several instructional techniques to educate the audience including the use of the songs, *Mommy is the best person in the world* and *Mommy, Grandma, and Aunties we love you, Please take good care of yourself*, which aimed to increase awareness of the women’s role in the family and highlight the innate care giving nature of women in the family which extends to the responsibility of the women to take care of themselves. In addition to the use of song, posters that presented breast cancer screening guidelines and educational messages from the Susan G. Komen Foundation were shared in both Chinese and English.

A baseline survey assessing knowledge of breast cancer screening guidelines, current screening practices, and age was distributed to audience members as they arrived. In the post-test, screening intentions, knowledge about breast screening guidelines and intentions to conduct a self-breast exam (SBE) and schedule an appointment for a clinical breast exam (CBE) and mammogram as recommended by the breast cancer screening guidelines were measured.

Results indicated that there was an increase in knowledge of breast cancer screening guidelines. For this largely Chinese immigrant population, the degree of acculturation and attentiveness to the play was positively associated with an increased knowledge score and the intent to follow the breast cancer screening guidelines. Approximately 46% of the participants reported that they would rather receive health messages incorporated into children’s performances than through conventional channels.

### Conference literature search

#### Types of studies presented at conferences

Eligibility criteria for conference abstracts included that they were in English; examined at least one-type of special event; and focused on breast, cervical or colorectal cancer prevention.

### Three-stage search and data gathering methodology for conference grey literature

#### Stage I: abstract search

From November 2011 to March 2012, the project team conducted a grey literature review of conference abstracts that occurred in the U.S., focused on breast, cervical and colorectal cancer and had screening-related outcomes. The advisory committee and the study team created a list and performed an internet search of national, regional, and local organizations that held conferences covered health promotion on cancer or breast, cervical, or colorectal cancer prevention.

Fourteen organizations that hosted eligible conferences were identified; however, 3 of these organizations (Prevent Cancer Foundation, National Colorectal Cancer Round Table, and National Cervical Cancer Coalition) were excluded because the conference abstracts were unavailable and 2 (Sisters Network Inc. and American Society of Preventive Oncology) were excluded for not focusing on breast, cervical or colorectal cancer prevention. The nine resulting organizations held a total of 23 conferences in the time period of interest (2009-2011) and 8,136 conference preceding abstracts were reviewed from those conferences (Table 2).

**Table 2:** Conferences reviewed

**Level**	**Name of organization**	**Years reviewed**	**# Abstracts reviewed**	**# Abstracts chosen for discussion**	**# Included studies**
World	World Conference on Breast Cancer Foundation	2011	122	1	0
National	Academy Health	2009-2011	445	0	N/A
National	Avon	2010	16	0	N/A
National	American Public Health Association (APHA)	2009-2011	4,330	15	3
National	National Association of County and City Health Officials (NACCHO)	2009-2011	131	0	N/A
National	National Institutes of Health (NIH) Colorectal Cancer Screening Conference	2010	22	0	N/A
National	Society for Public Health Education (SOPHE)	5/2009, 11/2009 4/2010, 11/2010 5/2011, 10/2011	605	0	N/A
National	Society of Behavioral Medicine (SBM)	2009-2011	2096	0	N/A
Regional	Cancer Prevention Research Institute of Texas (CPRIT)	2010 & 2011	369	4	2
	**Total**	**23 conferences**	**8,136**	**20**	**5**

Conference descriptions were independently reviewed by two study team members to determine if the conferences met study eligibility criteria. For a conference to be eligible, it had to be conducted between 2009 and 2011 in the United States and include topics on breast, cervical, and/or colorectal cancer prevention.

Once a conference was chosen for inclusion, the agendas and presentation and/or poster abstracts were obtained through the website or sponsor. If the website did not provide these materials then a sponsoring organization member, a conference attendee and/or a presenter collaborator was contacted to obtain this information for the review. Organizations for which we were not able to collect abstracts or conference agendas were excluded from the study (n = 3).

Two study team members independently reviewed the presentation and poster abstracts for study eligibility. Abstracts that did not provide enough information to determine eligibility were excluded for further review. This review resulted in 20 abstracts being chosen for group discussion with the principal investigator and project manager. Based on further examination of the cancer and event-type discussed in the abstract, 7 abstracts were excluded because of their alignment with the definition of a special event, the location of the event (excluded for being outside of the U.S.), and their screening focus.

#### Stage II: solicitation of additional information from presenters

The authors of the 13 included abstracts were contacted via e-mail or phone to request additional materials about the event(s) discussed in their presentation, including presentation slides, posters, printed public reports, or summaries. Only 7 participants responded with presentation and project information after six attempts.

Two trained reviewers abstracted the information that was provided by the presenters through the use of a Data Abstraction Form, a six-part form that included questions concerning the title, researcher(s), methods, results, reported cost of the event(s), resources, and barriers to implementation. After the reviewers completed abstraction of the additional materials, a second project team meeting was held to re-examine the eligibility of the conference presentations based on the additional information provided. Two conference presentations were excluded during this process. This resulted in five conference presentations included in Stage II.

#### Stage III: interviews with presenters for further information

The presenters, whose presentations were chosen for inclusion, were contacted a second time to set up a time to participate in a telephone interview to provide additional information on their event. A follow-up interview guide was created by the project team to better understand the event and included questions on the event’s type, goals and objectives, implementation steps, screening availability, partners, recruitment, costs, evaluation, follow-up, barriers, and other program components. Data that were already collected from the abstract or the other obtained materials were added to the interview guide prior to the interview to allow for verification or clarification during the interview. The telephone interviews were 30 to 45 minutes long and were recorded with consent from the presenter. One participant completed the interview guide through e-mail. Based on the data from the interview, the abstraction form for each conference presentation was completed.

#### Data obtained from the three-stage process

Table 3 shows what data were obtained at each stage, what data were newly obtained at each stage. Overall, much of the desired data was obtainable from the abstracts. However, the information provided in the abstracts varied in detail and many items such as types and sources of educational materials used and total costs were not included; other information such as implementation steps and recruitment data were absent from most abstracts. Data collected at stage 2 through author contact included information on partnerships (agencies involved), barriers, and study limitations for the majority of the studies. The phone interviews conducted in stage 3 allowed for the collection of data such as detailed cost information and more data regarding context. Overall, most of the data of interest was obtained through the three stage process; however, certain information such as screening, follow-up outcomes and total costs could not be obtained since they were not collected. Only 4 events presented post-screening data and 2 events had cost data.

**Table 3:** Type of conference data by method

**Type of conference data**	**Stage I: abstracts**	**Stage II: additional materials**	**Stage III: Interviews**
Author, Title, Conference, Date of Conference	5	5	0
**Study Design**			
Part of Additional Activities (Larger intervention or Research Project)	1	2	5
**Intervention**			
Goals and Objectives of Event or Research	4	5	4
Theory/Guiding Concept	5	4	2
Type of Event and Cancer	5	5	5
Type of Cancer Screening Services Provided (Exams, Referrals, Patient Navigators)	3	4	4
Community Guide Strategies Used	3	2	5
Other Health Services Provided	0	2	1
Other Health Topics Discussed	0	1	1
Educational Materials (sources, type)	0	2	5
**Delivery**			
Logistics (Date, Duration, Frequency)	4	5	1
Setting (Place, setting, area)	4	4	3
Who Provided Services	4	4	4
Agencies Involved (Host, Funders, Partners)	1	5	5
Implementation Steps	1	2	5
Target Population	5	5	0
Demographics of Participants	3	5	0
Recruitment	1	3	5
**Outcomes**			
Process Outcomes (If it was collected, results)	5	5	4
Screening Outcomes (If it was collected, results)	2	3	2
Follow-up (If it was collected, results)	1	3	5
Other Outcomes (If it was collected, results)	5	5	1
**Costs and Resources**			
Total Costs	0	0	3
Further Cost Information (Break-down of costs, cost analysis, resources)	0	1	5
**Discussion**			
Generalizability/Applicability	4	4	0
Barriers/Study Limitations	0	3	5
Recommendations	3	2	0

## Results

### Conferences

#### Description of the events presented at conferences

Our grey literature search of conference presentations resulted in identifying 5 programs that utilized 3 types of special events: 1) health fair [18], 2) reception/party/special meal [19-21], and 3) cultural event [22]. Details on each presentation’s target audience, implementation techniques, types of data collected, and results are described in Table 1. All of the programs focused on both breast and cervical cancer screening; no qualifying studies focused on colorectal cancer screening. All five of the programs were designed to target minority populations, including Latinas [18,22], Latina immigrants [19,21], African Americans [18,20], and the uninsured/underinsured [18-20].

Three of the programs involved standalone (single-component special event) events [19,20,22] while two programs performed events in conjunction with other outreach activities (multi-component) [18,21]. The other activity components that occurred in conjunction with the special events included canvassing, kiosks, hotlines, and referrals as part of the Helping Her Live Model [18] and activities such as media promotion for breast and cervical cancer screening, cultural competency training, and an end of year party as part of the Cuidandome intervention [21]. All of the events that reported their frequency occurred multiple times throughout their respective study periods, with a range of 14 [22] to 167 [21] events held during study periods ranging 7 months [20] to 7 years [15]. The cumulative number of participants at the events ranged widely from 880 [18] to 8,026 [22].

Activities at the events were categorized according to the Community Guide recommended strategies to increase cancer screening by the study team [8,9]. Four or more community guide strategies were utilized at all five of the events. The most commonly used strategies included group education, small media, and reducing structural barriers (employed at all 5 events), followed by reducing out-of-pocket costs (employed at 4 events), client reminders and recalls (employed at 2 events), and lastly, the use of mass media and one-on-one education (employed at 1 event). Common types of group education involved presentations from physicians, lay-health advisors, or cancer survivors [18-21]. Reducing out-of-pocket costs was done by providing access or resources for low-cost or free preventive screening services [19-22], while reducing structural barriers involved activities such as providing screening appointment sign-ups at the event [19,20] or providing transportation for event participants [18].

Screening data was collected post-event for all of the programs except one [20]. Pre and post-event data was collected at all five of the events. Mammogram screening rates ranged from 6.8% to 60% and Pap test from 52% to 70%. The denominator for screening reflects that number of attendees that the events reported in their event/study methods reported in the abstracts or interview data. Follow-up periods ranged from one-week to 15-months. Additionally, on-site clinical breast exams (CBE) were provided at events as part of one of the programs, which resulted in 3,158 (39%) women receiving a CBE [18]. Additional data that was collected during or after the event included screening determinants such as knowledge and intention to be screened [20,21] and barriers to care [22].

Finally, four of the programs provided additional cost information [18,20-22]. The type of information on program costs provided by participants varied. One event provided the total grant amount, two provided a number with limited description of what it represented, and the forth provided a per event cost break down. Based on the variety in the type of cost information collected, descriptive statistics could not be provided. One event of health parties detailed costs at $130 per event, while another of health forums had costs of $3000-$4,000 per event.

## Discussion

The grey literature review found 6 additional special events to add to our systematic review findings. Through our systemic review, we found five types of special events relevant to cancer screening: health fairs, parties, cultural events, special days, and a play. The types of events found in this grey literature review added to the numbers found in the systematic review, especially for receptions or parties and cultural festivals/events. The events all focused on increasing breast and cervical cancer screening except one that focused on breast cancer only. Similar to the systematic review findings, common evidence-based strategies recommended by the Guide to Community Preventive Services employed by these events were group education, small media and reducing structural barriers [8]. Five out of the six studies reported post event screening data [18,19,21,22]. The reach of these events was targeted to mostly minorities or underserved communities and large in terms of event participants. Many were offered multiple times [19-22]. The events did results in cancer screening; for the 4 events that reported screening, generally rates were higher for cervical cancer than breast cancer. However, there is limited data to support the overall effectiveness because the results found different categories of events, intervention components, and different cancer screenings reported. Special events could be opportunities to increase education and screening particularly if they are more regularly implemented in communities. In addition, they may also reach a segment of the population that may not have easy access to medical centers or health clinics and who attend special events to receive clinical services [23].

Combining a grey literature review with a systematic literature review did contribute to a greater understanding of special events for promoting cancer screening by enhancing both the breadth of events found and depth of knowledge of these events. Similar to another review of grey literature for medical interventions, we found more special events promoting cancer screening from conference abstracts [13]. Often, practitioners and researchers may share their work at professional meetings but may not publish their results in peer-reviewed journals. A systematic review of 11 studies of 39 meetings found that 31% of submitted abstracts both accepted and rejected to biomedical meetings were eventually published as full articles [24]. Barriers to subsequent publications from conference abstracts exist, including limits of time, a project or study is ongoing, a belief that the responsibility for writing a manuscript belonged to another co-author, funding and lack of participation from co-authors [25-27]. Therefore, similar to this study, the inclusion of grey literature is recommended when the intervention and outcomes are complex and have multiple components and in the situation that the volume of published literature is low [28].

Recently, CONSORT guidelines have been developed for conference organizers to provide specific instructions about the key elements of a trial that authors should report [29]. Although a common pitfall of conference abstracts is the limited amount of information that may be reported [30], this guidance will help professionals and researchers with some uniform reporting. However, for systematic reviews of interventions more detailed information may be needed especially in the characterization of the intervention beyond the essential features. For our study, contacting the presenters for additional materials such as Power Point presentations as well as a follow-up interview after locating conference abstracts helped augment the data offered in the abstract and could be recommended as a successful methodology to collect intervention data comparable to those from publications. We found more information about the special event interventions in the areas of intervention description such as behavioral change theories used, delivery (e.g., deliverers, implementation steps, recruitment methods), process evaluation data and cost information. Cost data is extremely important for the practice community since they would like to have cost analysis for different inventions in their decision making for intervention choices in addition to effectiveness [31]. This methodology of follow-up with authors has been documented as a strategy to obtain missing data found in articles in the conduct of systematic reviews, particularly through email [32] and should be recommended to enhance data obtained from abstracts of conferences for grey literature reviews. However, challenges to including grey literature, including additional research costs for further systematic data collection from unpublished sources and staff time, should be considered [28].

There are several limitations to this study. While the review team covered the spectrum of national cancer conferences with a practice focus, the conferences found were not comprehensive based on geography spread and years searched. This was true at the regional and local levels. Data were also limited by conferences that did not have past copies of proceedings or abstracts for review. The dissertation search was delimited in terms of being based in the US and its publication year. In addition, the team did not review internet sites or reports which are another source of grey literature [13]. In addition, we limited the searches to those sources only in English. Internet sites were not reviewed since the study team believed that they were less likely to have a rich description of one particular special event but were more likely to discuss them in general.

## Conclusions

This research adds to the knowledge about how special events are employed to promote and facilitate cancer screening. Further evaluation and dissemination of the outcomes of such events will help to prove their effectiveness, especially for specific types of events (e.g., health fairs). In addition, this study demonstrates the contribution of grey literature to systematic reviews in terms of yielding significantly more events and understanding of that type of intervention to complement published findings. It is recommended that for interventions often conducted in community settings to include a review of grey literature sources to expand the knowledge base of that intervention. Finally, for this study, we developed a systematic method for conducting a grey literature review based on a few guidance tools. For example, we decided to conduct interview to collect more specific data about the event in addition to the information provided in the conference abstract. More examination of how to conduct grey literature reviews should be published and elucidate areas such as sources for reviews, different methods for abstracting data from each source, and common indicators to track. While guidance on grey literature review exists, it is general and further research could help professionals interested in conducting a grey literature review [33]. Future research and evaluation of specific categories of special events (e.g., health fairs, parties/receptions) are needed to yield a greater understanding of the components and effectiveness of types of events that occur more commonly in communities.

## Competing interests

The authors declare that they have no competing interests.

## Authors’ contributions

CE conceived of the study. CE, KR, MK and RE participated in the design of the study and performed the data analysis. EP and MA helped with data collection, and all worked to draft the manuscript. All authors read and approved the final manuscript.

## Pre-publication history

The pre-publication history for this paper can be accessed here:

http://www.biomedcentral.com/1471-2407/14/454/prepub

## References

[B1] American Cancer Society Cancer facts & figures 2013 2013 Atlanta: American Cancer Society

[B2] Centers for Disease Control and Prevention Cancer screening–United States MMWR Morb Mortal Wkly Rep 2012 61 3 41 45 22278157

[B3] Siegel R Naishadham D Jemal A Cancer statistics 2012 CA Canc J Clin 2012 62 1 10 29 10.3322/caac.20138 22237781

[B4] Edwards BK Ward E Kohler BA Eheman C Zauber AG Anderson RN Jemal A Schymura MJ Lansdorp-Vogelaar I Seeff LC van Ballegooijen M Goede SL Ries LA Annual report to the nation on the status of cancer, 1975-2006, featuring colorectal cancer trends and impact of interventions to reduce future rates Cancer 2010 116 3 544 573 19998273 10.1002/cncr.24760 PMC3619726

[B5] Klabunde CN Vernon SW Nadel MR Breen N Seeff LC Brown ML Barriers to colorectal cancer screening: a comparison of reports from primary care physicians and average-risk adults Med Care 2005 43 9 939 944 16116360 10.1097/01.mlr.0000173599.67470.ba

[B6] Klabunde CN Schenck AP Davis WW Barriers to colorectal cancer screening among Medicare consumers Am J Prev Med 2006 30 4 313 319 16530618 10.1016/j.amepre.2005.11.006

[B7] George SA Barriers to breast cancer screening: an integrative review Health Care Women In 2000 21 1 53 65 10.1080/073993300245401 11022449

[B8] Sabatino SA Lawrence B Elder R Mercer SL Wilson KM DeVinney B Melillo S Carvalho M Taplin S Bastani R Rimer BK Vernon SW Melvin CL Taylor V Fernandez M Glanz K Community Preventive Services Task Force Effectiveness of interventions to increase screening for breast, cervical, and colorectal cancers: nine updated systematic reviews for the guide to community preventive services Am J Prev Med 2012 43 1 97 118 22704754 10.1016/j.amepre.2012.04.009

[B9] Baron RC Melillo S Rimer BK Coates RJ Kerner J Habarta N Chattopadhyay S Sabatino SA Elder R Leeks KJ Task Force on Community Preventive Services Intervention to increase recommendation and delivery of screening for breast, cervical, and colorectal cancers by healthcare providers: a systematic review of provider reminders Am J Prev Med 2010 38 1 110 117 20117566 10.1016/j.amepre.2009.09.031

[B10] Escoffery C Rodgers KC Kegler MC Haardörfer R Howard D Liang S Pinsker E Roland KB Allen J Ory MG Bastani R Fernandez ME Risendal BC Byrd T Coronado G A systematic review of special events to promote breast, cervical, and colorectal cancer screening in the United States BMC Publ Health 2014 14 274 10.1186/1471-2458-14-274 PMC3987802 24661503

[B11] Hart C Doing a literature search: a comprehensive guide for the social sciences 2001 London: Sage

[B12] Hopewell S McDonald S Clarke M Egger M Grey literature in meta-analyses of randomized trials of health care interventions Cochrane Database Syst Rev 2007 18 12 MR000010 17443631 10.1002/14651858.MR000010.pub3 PMC8973936

[B13] Higgins JPT Green S Cochrane handbook for systematic reviews of interventions version 5.1.0 2011 London: The Cochrane Collaboration [updated March 2011]

[B14] Lefebvre C Manheimer E Glanville JL Higgins JPT, Green S Chapter 6: Searching for studies Cochrane Handbook for Systematic Reviews of Interventions Version 5.1.0 2011 London: The Cochrane Collaboration

[B15] Scherer RW Langenberg P von Elm E Full publication of results initially presented in abstracts Cochrane Database Syst Rev 2007 18 2 MR000005 17443628 10.1002/14651858.MR000005.pub3

[B16] Dobbins M Robeson P A methodology for searching the grey literature for effectiveness evidence syntheses related to public health 2006 Ottawa: The Public Health Agency of Canada

[B17] Sun A Promoting breast cancer screening among Chinese American women through young children's theatrical performance PhD thesis 2009 Minneapolis MN: Walden University

[B18] Hunt B Vasquez-Jones G Taylor C Rodriguez W Jimenez N Shah A Reaching at-risk women to promote breast cancer screening on the westside of Chicago 2011 Washington DC: Sinai Urban Health Institute

[B19] White K Garces I McGuire A Isabel S Does conducting routine community health outreach events reach new women: An evaluation of a breast and cervical cancer screening program for Latina immigrant women 2010 Denver: University of Alabama

[B20] Rice C Kirk A Talwar D Wood R Ory M Implementation of an Evidence-Based Cancer Prevention and Control Program through Texas AgriLife Extension: Friend to Friend 2011 Austin: Texas Agrilife Extension Service

[B21] Vera-Cala L Martinez-Donate A Vedro R Angulo R Atkinson T Effectiveness of Cuidandome (Taking Care of Me): a communitywide intervention to promote breast and cervical cancer screening among low-acculturated Latinas in Dane County, Wisconsin 2011 Washington DC: University of Wisconsin at Madison

[B22] Dahlke DV Gines V Villarreal S Ory M Increasing screening rates for Latinas using health fiestas and promotoras 2011 Houston: Texas A&M Health Science Center. Cancer Prevention and Research Institute of Texas Conference

[B23] Brouwers MC De Vito C Bahirathan L Carol A Carroll JC Cotterchio M Dobbins M Lent B Levitt C Lewis N McGregor SE Paszat L Rand C Wathen N Effective interventions to increase the uptake of breast, cervical and colorectal cancer screening: an implementation guideline Implement Sci 2011 6 112 21958602 10.1186/1748-5908-6-112 PMC3222606

[B24] Macias EP Morales LS Utilization of health care services among adults attending a health fair in south Los Angeles County J Comm Health 2000 25 1 35 46 10.1023/a:1005188801228 PMC1781363 10706208

[B25] von Elm E Costanza MC Walder B Tramèr MR More insight into the fate of biomedical meeting abstracts: a systematic review BMC Med Res Methodol 2003 3 12 12854973 10.1186/1471-2288-3-12 PMC184388

[B26] Sprague S Bhandari M Devereaux PJ Swiontkowski MF Tornetta P Cook DJ Dirschl D Schemitsch EH Guyatt GH Barriers to full-text publication following presentation of abstracts at annual orthopaedic meetings J Bone Joint Surg Am 2003 85-A 158 163 12533587 10.2106/00004623-200301000-00024

[B27] Krzyzanowska MK Pintilie M Tannock IF Factors associated with failure to publish large randomized trials presented at an oncology meeting JAMA 2003 290 4 495 501 12876092 10.1001/jama.290.4.495

[B28] Benzies KM Premji S Hayden KA Serrett K State-of-the-evidence reviews: advantages and challenges of including grey literature Worldviews Evid Based Nurs 2006 3 2 55 61 17040510 10.1111/j.1741-6787.2006.00051.x

[B29] Hopewell S Clarke M How completely are trials reported? Clin Trials 2005 2 3 265 268 16279150 10.1191/1740774505cn091oa

[B30] Hopewell S Clarke M Moher D Wager E Middleton P Altman DG Schulz KF Consort Group CONSORT for reporting randomized trials in journal and conference abstracts Lancet 2008 371 281 283 18221781 10.1016/S0140-6736(07)61835-2

[B31] Hannon PA Fernandez ME Williams RS Mullen PD Escoffery C Kreuter MW Pfeiffer D Kegler MC Reese L Mistry R Bowen DJ Cancer control planners' perceptions and use of evidence-based programs J Publ Health Manag Pract 2010 16 3 E1 E8 10.1097/PHH.0b013e3181b3a3b1 PMC2920604 20357600

[B32] Young T Hopewell S Methods for obtaining unpublished data Cochrane Database Syst Rev 2011 11 MR000027 22071866 10.1002/14651858.MR000027.pub2 PMC7390448

[B33] Moher D Liberati A Tetzlaff J Altman DG PRISMA Group Preferred reporting items for systematic reviews and meta-*a*nalyses Ann Intern Med 2009 151 4 W64 10.7326/0003-4819-151-4-200908180-00135 19622511

